# Use of Cells Expressing γ Subunit Variants to Identify Diverse Mechanisms of AMPK Activation

**DOI:** 10.1016/j.cmet.2010.04.001

**Published:** 2010-06-09

**Authors:** Simon A. Hawley, Fiona A. Ross, Cyrille Chevtzoff, Kevin A. Green, Ashleigh Evans, Sarah Fogarty, Mhairi C. Towler, Laura J. Brown, Oluseye A. Ogunbayo, A. Mark Evans, D. Grahame Hardie

**Affiliations:** 1 Division of Molecular Physiology, College of Life Sciences, University of Dundee, Dundee DD1 5EH, Scotland, UK; 2 Centre for Integrative Physiology, College of Medicine and Veterinary Medicine, University of Edinburgh, Edinburgh EH8 9XD, Scotland, UK

**Keywords:** HUMDISEASE, SIGNALING

## Abstract

A wide variety of agents activate AMPK, but in many cases the mechanisms remain unclear. We generated isogenic cell lines stably expressing AMPK complexes containing AMP-sensitive (wild-type, WT) or AMP-insensitive (R531G) γ2 variants. Mitochondrial poisons such as oligomycin and dinitrophenol only activated AMPK in WT cells, as did AICAR, 2-deoxyglucose, hydrogen peroxide, metformin, phenformin, galegine, troglitazone, phenobarbital, resveratrol, and berberine. Excluding AICAR, all of these also inhibited cellular energy metabolism, shown by increases in ADP:ATP ratio and/or by decreases in cellular oxygen uptake measured using an extracellular flux analyzer. By contrast, A769662, the Ca^2+^ ionophore, A23187, osmotic stress, and quercetin activated both variants to varying extents. A23187 and osmotic stress also increased cytoplasmic Ca^2+^, and their effects were inhibited by STO609, a CaMKK inhibitor. Our approaches distinguish at least six different mechanisms for AMPK activation and confirm that the widely used antidiabetic drug metformin activates AMPK by inhibiting mitochondrial respiration.

## Graphical abstract

**Graphical Abstract:**

**Figure:**
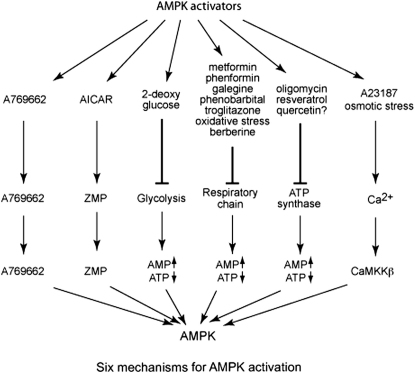

**Highlights:**

► AMPK is activated by a wide variety of different stresses, drugs, and xenobiotics ► We have developed a sensitive method to test which activators are AMP mediated ► Most, e.g., metformin and resveratrol, inhibit mitochondrial function and elevate AMP ► However, we can now distinguish six different mechanisms for AMPK activation

## Introduction

The AMP-activated protein kinase (AMPK) is a sensor of cellular energy status comprising heterotrimers formed from a catalytic α subunit that is only active after phosphorylation at Thr-172 by upstream kinases, and regulatory (β and γ) subunits (Hardie, 2007). There are several alternative upstream kinases in mammals, including the tumor suppressor LKB1 (Hawley et al., 2003; Woods et al., 2003) and the calmodulin-dependent kinase kinases CaMKK-α and -β (Hawley et al., 2005; Hurley et al., 2005; Woods et al., 2005). Any cellular stress that depletes cellular ATP and increases the ADP:ATP ratio causes, via adenylate kinase, a much larger increase in the AMP:ATP ratio. AMP binding stimulates AMPK by two mechanisms: (1) allosteric activation of phosphorylated AMPK, and (2) inhibition of dephosphorylation of Thr-172 by protein phosphatases (Davies et al., 1995; Suter et al., 2006; Sanders et al., 2007b). Both effects are caused by binding of AMP at two exchangeable sites on the γ subunits and are opposed by ATP, which binds at the same sites (Scott et al., 2004; Xiao et al., 2007). LKB1 provides a high basal Thr-172 phosphorylation, against which the inhibitory effect of AMP on dephosphorylation can operate. Activation of CaMKKs by Ca^2+^/calmodulin provides an alternate pathway by which AMPK can be activated by increasing intracellular Ca^2+^ levels, independently of AMP.

Once activated, AMPK switches on catabolic pathways that generate ATP, while switching off ATP-requiring processes not essential for short-term cell survival, including cell growth and proliferation (Hardie, 2007). Numerous stresses activate AMPK, including treatment with heat shock or metabolic poisons (Corton et al., 1994), muscle contraction (Winder and Hardie, 1996), glucose deprivation (Salt et al., 1998), hypoxia (Evans et al., 2005), oxidative stress (Choi et al., 2001), and osmotic stress (Fryer et al., 2002). AMPK is also regulated by cytokines involved in regulating whole-body energy balance (Kahn et al., 2005). In addition, a bewildering variety of drugs and xenobiotics has been found to activate AMPK, including (1) A-769662, developed by Abbott laboratories (Cool et al., 2006); (2) the antidiabetic biguanides, metformin (Zhou et al., 2001) and phenformin (Hawley et al., 2003), and a related natural product, galegine (Mooney et al., 2008); (3) another class of antidiabetic drug, the thiazolidinediones, (Fryer et al., 2002); (4) the barbiturate, phenobarbital (Rencurel et al., 2005); and (5) phytochemicals derived from traditional medicines, such as berberine (Lee et al., 2006), or present in foods or beverages, such as quercetin (Ahn et al., 2008; Suchankova et al., 2009), resveratrol (Baur et al., 2006), genistein, capsaicin, and epigallocatechin gallate (Choi et al., 2001; Hwang et al., 2005).

It is a challenge to understand how such a variety of compounds with different structures all lead to AMPK activation, and various suggestions have been made. Biguanides (El-Mir et al., 2000; Owen et al., 2000) and thiazolidinediones (Brunmair et al., 2004) are inhibitors of complex 1 of the respiratory chain, indicating that they might activate AMPK via inhibition of ATP synthesis and consequent increases in AMP. However, while thiazolidinediones (Fryer et al., 2002; Lebrasseur et al., 2006) and phenformin (Hawley et al., 2005) have been reported to alter adenine nucleotide ratios in cultured cells, activation by metformin was seen under conditions where no such changes were detectable (Fryer et al., 2002; Hawley et al., 2002). Similarly, osmotic stress (Fryer et al., 2002), hydrogen peroxide (Choi et al., 2001), and resveratrol (Suchankova et al., 2009) have been claimed to activate AMPK without any change in cellular nucleotides.

The AMPK system is exquisitely sensitive to small changes in AMP (Hardie et al., 1999; Suter et al., 2006), and it seemed to us that it might be activated by subtle changes in cellular nucleotides that may not be detectable by measuring total cellular levels. Adenine nucleotides are compartmentalized between the mitochondria and cytoplasm and, due to limitations on diffusion, may not be uniformly distributed even within the cytoplasm (Dzeja and Terzic, 2003). Cellular concentrations of ADP and AMP are usually too low to be measured directly by ^31^P NMR; their contents can be estimated in cell extracts, but in that case effects of compartmentation will be lost. Therefore, while an agent like metformin may activate AMPK without producing a detectable change in adenine nucleotides, this does not prove that the effect is AMP independent. We felt that it was important to develop a more sensitive diagnostic test to determine whether or not activation of AMPK by different agents was AMP dependent. The recent discovery of mutations that render the AMPK complex AMP insensitive provided an opportunity to achieve this. Point mutations in the γ2 subunit are associated with elevated glycogen in cardiac myocytes, leading to cardiac arrhythmias and hypertrophy (Arad et al., 2007). These mutations interfere with the binding of the regulatory nucleotides, AMP and ATP, to two sites on the γ subunit (Scott et al., 2004; Xiao et al., 2007). For the present study, we have selected one of these mutations, R531G, that causes a severe loss of binding of AMP and ATP, thus generating an AMP-insensitive complex (Scott et al., 2004). We constructed isogenic HEK293 cells stably expressing either wild-type γ2 or this mutant and used them to test whether a variety of pharmacological agents and stresses that activate AMPK do so via increases in AMP.

## Results

### Characterization of Stably Transfected Cells

**Figure 1:**
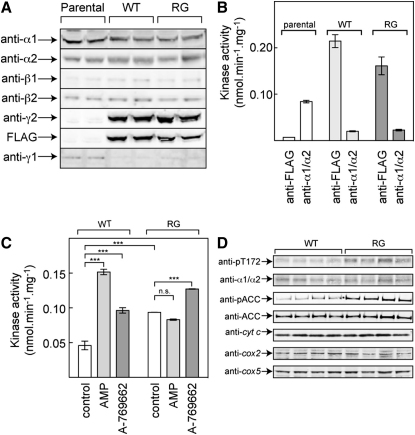
Analysis of HEK293 Cells Stably Expressing WT γ2 and the R531G Mutant (A) Duplicate western blots of parental HEK293 cells and WT or RG cells using antibodies against the α1, α2, β1, β2, γ1, and γ2 subunits of AMPK, and against the FLAG epitope on γ2. (B) AMPK activities in parental HEK293 cells and WT and RG cells, in samples made by immunoprecipitation using anti-FLAG antibodies, and by subsequent immunoprecipitation from the anti-FLAG supernatants using anti-α1/α2 antibodies. Results (mean ± SD, n = 2) are expressed as units per milligram of protein in the volume of cell lysate from which the first immunoprecipitation was performed. (C) Effect of AMP (200 μM) and A769662 (1 μM) in cell-free assays of anti-FLAG immunoprecipitates from WT and RG cells. Results expressed as in (B). Significant differences between indicated groups were determined by one-way ANOVA (^∗∗∗^p < 0.001; n.s., not significant). (D) Western blots (n = 4) showing phosphorylation (pT172) and expression (α1/α2) of AMPK, phosphorylation (pACC) and expression (ACC) of acetyl-CoA carboxylase, and expression of three mitochondrial markers (*cyt c*, cytochrome c; *cox2*, flavoprotein subunit of succinate dehydrogenase [SDHA]; *cox5*, ATP synthase, F_1_ complex, β subunit) in WT and RG cells.

We generated stable, isogenic HEK293 lines expressing the WT or the R531G mutant using the Flp-In system, in which the DNA is inserted by homologous recombination into a single Flp recombination target (FRT) site previously introduced into the host cells. The α, β, and γ subunits of AMPK are unstable when expressed individually in cells, and coexpression of all three is necessary to obtain significant overexpression. We therefore suspected that if we stably expressed only the γ2 subunit, it might replace the endogenous γ1 subunit in the HEK293 cells, a hypothesis supported by the data in Figure 1. By Western blotting (Figure 1A), the expression of the α1, α2, β1, and β2 subunits was not significantly altered in cells stably expressing wild-type γ2 (referred to below as WT cells) or the R531G mutant (RG cells), compared with the parental cells containing the FRT site. As expected, γ2 was readily detectable by Western blotting in the WT and RG cells, using either anti-γ2 or anti-FLAG antibodies. However, expression of the endogenous γ1 subunit decreased markedly, suggesting that the recombinant γ2 had largely, if not entirely, replaced it. This was confirmed by another approach (Figure 1B). When cell extracts were immunoprecipitated with anti-FLAG antibodies, the activity recovered from the parental cells was negligible as expected, but immunoprecipitation from the resulting supernatant using anti-α subunit antibodies yielded a substantial activity, corresponding to the endogenous AMPK complexes containing γ1. If the same experiments were carried out with WT or RG cells, activities 2- to 3-fold higher than the endogenous activity of the parental cells (measured in the presence of AMP) were recovered using anti-FLAG antibody, but the activity recovered from the subsequent immunoprecipitate using anti-α antibodies was reduced by > 80%. These results show that the recombinant FLAG-tagged γ2 subunit had largely replaced the endogenous γ1 subunit within AMPK complexes in the WT and RG cells.

We next examined the effects of AMP and A769662 in cell-free assays of AMPK recovered using anti-FLAG antibodies from WT and RG cells. AMPK from WT cells was activated 3-fold by AMP and 2-fold by A769662 (Figure 1C). As expected, AMPK from RG cells was not activated by AMP, but it was still stimulated by A769662, consistent with previous results (Sanders et al., 2007a) showing that the equivalent R298G mutation in γ1 did not affect activation by A769662. Another notable finding in Figure 1C was that the basal activity (measured in the absence of AMP) of the R531G mutant was 2-fold higher (p < 0.001) than that in the WT cells. This increased activity correlated with a 2-fold higher level of phosphorylation of AMPK at Thr-172 (pT172, p < 0.001), and a 2.6-fold increase in phosphorylation of acetyl-CoA carboxylase (pACC, p < 0.0001), with no change in expression of either protein (Figure 1D).

### Effects of Metabolic Stresses and AMPK Activators

**Figure 2:**
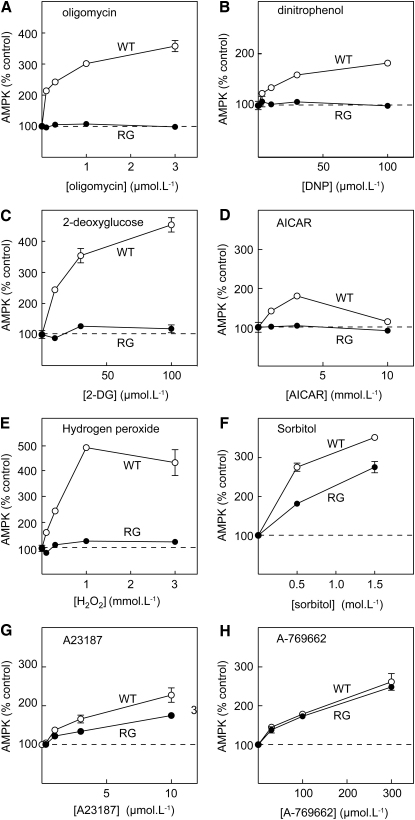
Concentration Dependence of AMPK Activation by Various Agents Results are shown for WT cells (open circles) or RG cells (filled circles). Incubations with the indicated agent were for 1 hr, after which AMPK was immunoprecipitated using anti-FLAG antibody and assayed at 200 μM AMP. Results (mean ± SD, n = 2) are expressed as percentages of the activity in controls without added agent.

We next tested on WT and RG cells various agents previously shown to activate AMPK (Figure 2). Oligomycin (an inhibitor of the mitochondrial ATP synthase), dinitrophenol (DNP, which uncouples the respiratory chain from ATP synthesis), 2-deoxyglucose (an inhibitor of glycolysis), 5-aminoimidazole-4-carboxamide riboside (AICAR, a nucleoside converted within the cell to an AMP mimetic), and the reactive oxygen species, hydrogen peroxide, all activated AMPK in WT cells but had no effect in RG cells. By contrast, the effects of osmotic stress (induced by the cell-impermeant solute, sorbitol) and increasing cytoplasmic calcium using the Ca^2+^ ionophore, A23187, were reduced but not abolished in RG cells. Finally, activation by A769662 was completely unaffected by the RG mutation. These latter results provide important positive controls, because they show that the RG mutant, although insensitive to AMP, is nevertheless functional and can still be activated by treatments that do not involve a change in cellular AMP.

### Effects of Other AMPK-Activating Drugs

**Figure 3:**
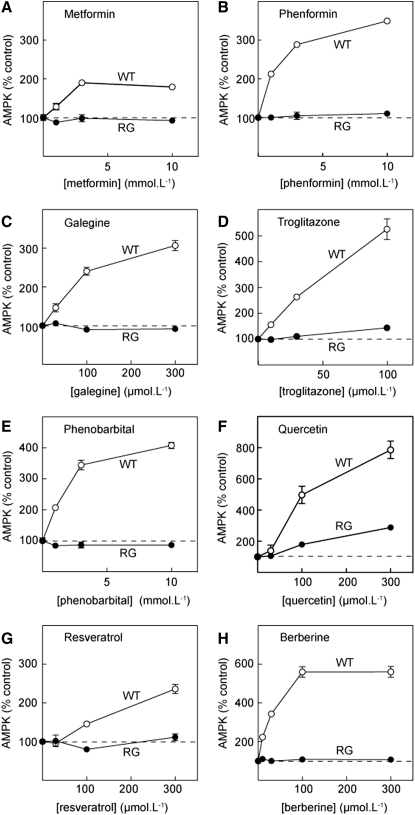
Concentration Dependence of AMPK Activation by the Indicated Drugs and Phytochemicals Results are shown for WT cells (open circles) or RG cells (filled circles). Incubations with the indicated agent were for 1 hr (except metformin, 16 hr), after which AMPK was assayed as for Figure 2. Results (mean ± SD, n = 2 except quercetin, n = 4) are expressed as percentages of the activity in controls without added agent.

We next tested the effects of a variety of drugs and xenobiotics that have been reported to activate AMPK in cell-based assays (Figure 3). First, we studied biguanides currently or previously used to treat type 2 diabetes, i.e., metformin and phenformin. Metformin uptake in HEK293 cells is very slow due to lack of expression of the transporter OCT1 (Shu et al., 2007), so incubation with metformin was for 16 hr rather than the 1 hr incubation used for all other treatments. Nevertheless, metformin and phenformin, as well as the related natural product galegine (isoprenyl guanidine), clearly activated AMPK in WT cells, but not RG cells. Similar results were obtained with the thiazolidinedione, troglitazone, and the barbiturate, phenobarbital. Finally, we studied quercetin (a plant flavonoid), resveratrol (a polyphenol present in red wine), and berberine (from the genus *Berberis*, used in traditional Chinese medicine). All three agents caused a robust activation of AMPK in WT cells. Resveratrol and berberine had no effect in RG cells, although a modest effect did remain with quercetin.

### Phosphorylation of AMPK

**Figure 4:**
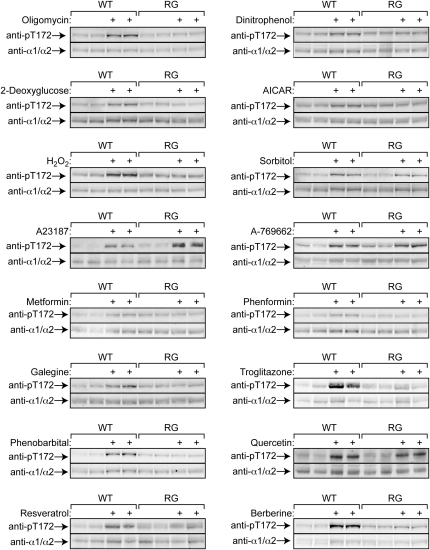
Phosphorylation of Thr-172 on AMPK-α in WT or RG Cells in Response to Various Agents Cells were incubated in duplicate for 1 hr (except metformin, 16 hr) with the indicated agent at the following concentrations: oligomycin (1 μM), DNP (100 μM), 2-deoxyglucose (30 mM), AICAR (3 mM), hydrogen peroxide (1 mM), sorbitol (1.5 M), A23187 (3 μM), A769662 (300 μM), metformin (3 mM), phenformin (3 mM), galegine (100 μM), troglitazone (100 μM), phenobarbital (3 mM), quercetin (100 μM), resveratrol (300 μM), and berberine (100 μM).

The assays in Figures 2 and 3 were conducted in immunoprecipitates at saturating AMP concentration (200 μM), suggesting that the effects were due to increased phosphorylation rather than allosteric activation. Confirming this, there was a good correlation between increased AMPK activation and Thr-172 phosphorylation assessed by Western blotting. Thus, all 16 treatments produced clear increases in Thr-172 phosphorylation in WT cells without any changes in α1/α2 subunit expression (Figure 4). With oligomycin, DNP, 2-deoxyglucose, AICAR, hydrogen peroxide, metformin, phenformin, galegine, troglitazone, phenobarbital, resveratrol, and berberine, this was only observed in WT but not RG cells, while with sorbitol, A23187, A769662, and quercetin there was also increased Thr-172 phosphorylation in RG cells.

### Changes in Cellular Nucleotides

**Figure 5:**
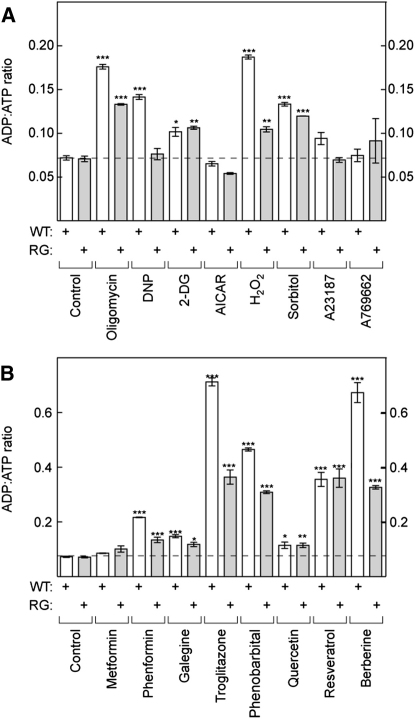
Effect of Indicated Treatments on ADP:ATP Ratios in WT or RG Cells Cells were incubated for 1 hr (except metformin, 16 hr) with the indicated agent at concentrations used in Figure 4. Contents of ATP and ADP in perchloric acid extracts were estimated using capillary electrophoresis and are expressed as a ratio. Results are mean ± SD (n = 16 for controls; n = 2 for all others). Means that are significantly different by one-way ANOVA from controls performed at the same time without treatment are indicated: ^∗^p < 0.05, ^∗∗^p < 0.01, ^∗∗∗^p < 0.001. Abbreviations: DNP, dinitrophenol; 2-DG, 2-deoxyglucose.

If treatments that activate AMPK in WT but not RG cells do so because they increase cellular AMP, they would be predicted to alter cellular nucleotide contents. This was addressed using capillary electrophoresis to measure nucleotides in perchloric acid extracts. While AMP could usually be detected in treated cells (data not shown), its content was too low to reliably estimate in the untreated cells. To obtain a quantitative measure of energy stress, we therefore measured the ADP:ATP ratio and used it as a surrogate for AMP:ATP (which, assuming that adenylate kinase reaction is at equilibrium, will vary as the square of ADP:ATP [Hardie and Hawley, 2001]). As expected, the mitochondrial inhibitors oligomycin and DNP, and the glycolytic inhibitor 2-deoxyglucose, all increased the ADP:ATP ratio in WT cells (Figure 5A), whereas AICAR, A23187, and A769662 did not produce a significant change. Hydrogen peroxide increased the ADP:ATP ratio significantly in WT cells at 0.3 mM and to a much greater extent (15-fold, data not shown) at 1 mM. Osmotic stress using sorbitol also caused a small increase in ADP:ATP in both WT and RG cells.

Results with a wide range of AMPK-activating drugs and xenobiotics are shown in Figure 5B. Although metformin appeared to cause small increases in ADP:ATP (16% in WT and 38% in RG cells), these were not statistically significant. All other drugs produced significant increases, both in WT and RG cells. The effect of 100 μM quercetin was small but significant (1.6-fold, Figure 5B), while the effect of 300 μM quercetin (data not shown) was much larger (18-fold in WT and 33-fold in RG cells).

Interestingly, with the exceptions of 2-deoxyglucose, quercetin, and resveratrol, the agents that caused increases in the ADP:ATP ratio tended to cause larger increases in the WT than in the RG cells, suggesting that the latter might be more resistant to metabolic stress. Since AMPK has been found to promote mitochondrial biogenesis, we initially hypothesized that the higher basal AMPK activity in the RG cells (Figure 1) meant that they might have higher mitochondrial content and thus a greater capacity to generate ATP. However, this appears not to be the case. First, expression of a number of mitochondrial markers (*cyt c*, *cox2*, *cox5*) was not different between the WT and RG cells (Figure 1D). Second, the density of mitochondria by fluorescence microscopy of cells stained with MitoTracker dye was not different (data not shown). Third, the rate of oxygen uptake in WT and RG cells, when maximally stimulated using DNP, was not significantly different (see next section).

### Most Treatments that Increase AMP Inhibit O_2_ Uptake

To confirm that most treatments that activate AMPK in a strictly AMP-dependent manner act by inhibiting mitochondrial metabolism, we analyzed their effects on oxygen uptake using an extracellular flux analyzer. Basal oxygen uptake was very similar between the two cell lines (WT, 4.6 ± 1.3 [mean ± SD, n = 38]; RG, 4.3 ± 0.8 [n = 31] nmol/min/10^6^ cells). If the cells were titrated with an inhibitor of the ATP synthase, oligomycin, oxygen uptake in the WT cells decreased by a maximum of 60% to 1.8 ± 0.4 (WT, n = 7) and 1.6 ± 0.2 (RG, n = 8) nmol/min/10^6^ cells. If the cells were titrated with the uncoupler DNP, basal oxygen uptake in the WT cells increased by a maximum of ≈3-fold to 15.4 ± 2.1 (WT, n = 5) and 13.7 ± 2.3 (RG, n = 8) nmol/min/10^6^ cells. The rates in the presence of DNP represent estimates of the maximal rate of the respiratory chain. Neither the basal, the oligomycin-inhibited, nor the DNP-stimulated rates were significantly different between the WT and RG cells, confirming that the mitochondrial capacities of these cells were similar.

**Figure 6:**
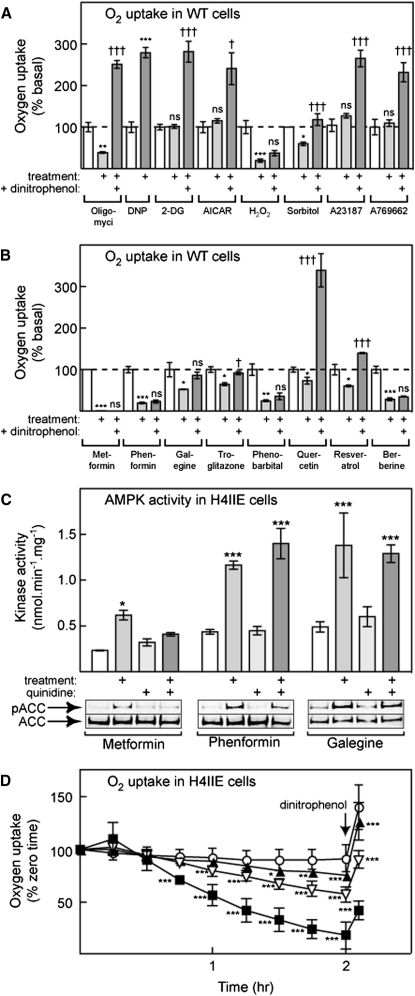
Effect of Various Treatments on Oxygen Uptake and AMPK Activity in HEK293 and H4IIE Cells (A and B) Effects of different treatments on oxygen uptake in WT HEK293 cells. Results are mean ± SEM, with n = from 4 to 10. Concentrations of the indicated agent were as in Figure 4. Except in the case of metformin and sorbitol, oxygen uptake was measured in the same well before treatment (basal), after treatment for 1 hr, and after subsequent addition of DNP. Statistical significance was assessed using repeated-measures ANOVA; differences between basal and treatment: ^∗^p < 0.05; ^∗∗^p < 0.01; ^∗∗∗^p < 0.001; differences between treatment and treatment plus DNP: ^†^p < 0.05; ^††^p < 0.01; ^†††^p < 0.001. In the case of metformin and sorbitol, technical issues prevented a repeated-measures design. Instead, oxygen uptake in treated wells (16 hr metformin, 1 hr sorbitol) was compared with control wells on the same plate, although the effect of DNP was compared in the treated well. Statistical significance of differences between untreated and treated wells was by unpaired t test. (C) Activation of AMPK (top), and phosphorylation of the AMPK target, ACC, (bottom) in H4IIE cells treated for 1 hr with 3 mM metformin, 300 μM phenformin, or 30 μM galegine with or without pretreatment (15 min) with 10 μM quinidine. Data are mean ± SD (n = 2). Statistical significance was assessed using two-way ANOVA; significant differences between drug-treated samples and controls are indicated: ^∗^p < 0.05, ^∗∗∗^p < 0.001. (D) Oxygen uptake in H4IIE cells treated without metformin (open circles) or with 1 mM (filled triangles), 3 mM (open inverted triangles), or 10 mM metformin (filled squares) for the times indicated. Data are mean ± SD (n = 15). Statistical significance was assessed using two-way ANOVA; significant differences between control and drug-treated samples at the same time point are indicated: ^∗^p < 0.05, ^∗∗^p < 0.01, ^∗∗∗^p < 0.001.

All stress and drug treatments also had very similar effects on oxygen uptake in WT cells (Figures 6A and 6B) and in RG cells (see Figure S1 available online). Hydrogen peroxide, phenformin, galegine, troglitazone, Phenobarbital, and berberine all inhibited basal oxygen uptake in a similar manner, and in these cases there was no significant stimulation of oxygen uptake on subsequent addition of DNP, indicating that they were inhibiting the respiratory chain. Osmotic stress also reduced oxygen uptake; subsequent addition of DNP increased the rate slightly, but not to the level seen after oligomycin. Resveratrol and quercetin inhibited basal oxygen uptake, although with quercetin inhibition was not significant at 100 μM and was only modest (25%, Figure 6 and Figure S1) at 300 μM. With these agents there was a large stimulation in oxygen uptake after subsequent addition of DNP, although with resveratrol this did not reach the level seen after oligomycin or quercetin. 2-deoxyglucose, AICAR, A23187, and A769662 produced no changes either in basal or DNP-stimulated oxygen uptake.

Metformin appeared to produce an almost total inhibition of oxygen uptake with no subsequent stimulation by DNP (Figure 6B). However, because of the slow uptake of metformin by HEK293 cells, the incubation with metformin was for 16 hr rather than 1 hr as for other treatments. Due to the long incubation we were also forced to use a different experimental design in which the basal and metformin-inhibited rates were measured in different wells on the same plate, rather than in the same wells before and after addition of the drug as for other treatments. To circumvent the problem of the slow uptake of metformin by HEK293 cells, we also studied H4IIE cells, a rat hepatoma cell line that we suspected might express the organic cation tranporter OCT1, which promotes metformin uptake (Shu et al., 2007). In these cells, activation of endogenous AMPK by metformin was much more rapid, reaching a maximum after 2–3 hr with a half-maximal effect at 1.1 mM (data not shown), consistent with a reported Km of OCT1 for metformin of 1.5 mM (Koepsell et al., 2007). To confirm that the more rapid effect of metformin in H4IIE cells was due to the expression of OCT1, we investigated the effects of quinidine, a high-affinity substrate for OCT1 that acts as a competitive inhibitor. As expected, in H4IIE cells quinidine almost completely blocked AMPK activation, as well as the phosphorylation of the AMPK target, ACC, by metformin. By contrast, quinidine had no effect on the responses to phenformin or galegine (Figure 6C). Figure 6D shows that, after a lag of about 30 min, metformin caused a time- and concentration-dependent decrease of oxygen uptake in H4IIE cells. At 10 mM metformin, inhibition of oxygen uptake was significant by 45 min and had reached > 80% by 2 hr. After this, addition of a maximally effective concentration of the uncoupler DNP caused only a modest stimulation of oxygen uptake, consistent with the idea that metformin was inhibiting the respiratory chain.

### Effect of STO-609 and Ca^2+^ Measurements

**Figure 7:**
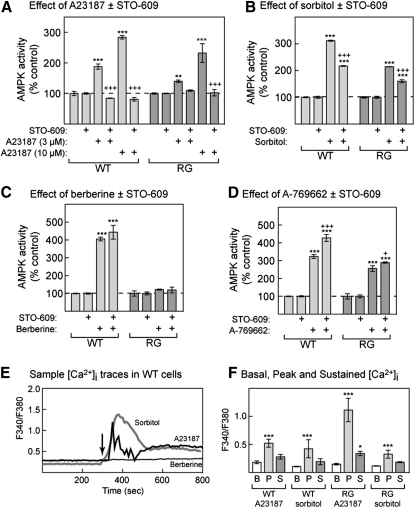
Effect of the CaMKK Inhibitor STO-609 on AMPK Activation by A23187, Sorbitol, Berberine, and A769662; and Effect of A23187, Sorbitol, and Berberine on Intracellular Ca^2+^ (A–D) WT or RG cells were incubated with the following: (A) A23817 (3 or 10 μM), (B) sorbitol (1.5 M), (C) berberine (100 μM), and (D) A769662 (300 μM), with or without 10 μM STO-609 for 1 hr. AMPK was assayed as in Figure 2. Results are expressed as a percentage of the average activity in control cells as mean ± SD (n = 2). Statistical significance was assessed by two-way ANOVA (significantly different from control without primary agent (^∗∗∗^p < 0.001) or without STO-609 (^+^p < 0.05, ^+++^p < 0.001). (E) Records of Fura-2 fluorescence ratio (F_340_/F_380_) against time illustrating the effect of 10 μM A23187, 0.15 M sorbitol, or 30 μM berberine on cytoplasmic Ca^2+^ concentration in individual WT cells. Each agent was added at 300 s (arrow). (F) Average F_340_/F_380_ values for the basal (B, prior to treatment), peak (P), and sustained (S) phases of response to 10 μM A23187 and 0.15 M sorbitol in WT and RG cells. Data are mean ± SD (n = from 3 to 7. Significance of differences from basal was assessed by two-way ANOVA: ^∗^p < 0.05, ^∗∗∗^p < 0.001.

To examine whether activation of AMPK by agents that acted in an AMP-independent manner required calmodulin-dependent protein kinase kinases (CaMKKs), we utilized the CaMKK inhibitor, STO-609 (Figure 7A). As expected, increasing concentrations (3 and 10 μM) of the Ca^2+^ ionophore A23187 progressively increased AMPK activity, and the effect of either concentration was completely blocked by STO-609. The results were essentially identical with WT or RG cells. The effects of osmotic stress using sorbitol were also inhibited significantly, but not completely, by STO-609 in both cell types (Figure 7B), suggesting that in this case the effect is only partially mediated by CaMKKs. As expected, STO-609 did not inhibit activation of AMPK by berberine or A769662 (Figures 7C and 7D).

We also compared the effects of A23187, sorbitol, and berberine on cytoplasmic Ca^2+^ concentration assessed using the Ca^2+^-sensitive fluorescent dye, fura-2. Example time courses in individual WT cells are shown in Figure 7E, while results of several experiments in WT and RG cells are summarized in Figure 7F. Within 1 min of addition, A23187 and sorbitol produced large but transient increases in intracellular Ca^2+^ in both WT and RG cells. The Ca^2+^ levels then declined to a level that remained above the baseline for at least 8 min. There was no detectable change in intracellular Ca^2+^ concentration during incubation for up to 10 min following addition of 30 μM berberine, in either cell line (n = 6).

## Discussion

Our stable cell lines expressing the wild-type and R531G mutant versions of γ2 provide a sensitive assay to test whether agents that activate AMPK do so via AMP-dependent or AMP-independent mechanisms. A769662 (a direct AMPK activator unaffected by the R531G mutation) and the Ca^2+^ ionophore A23187 (which increases cytoplasmic Ca^2+^ and thus activates the upstream kinase, CaMKKβ) caused increased phosphorylation and activation of AMPK in both WT and RG cells, showing that the R531G mutant is still capable of being activated, and providing important positive controls. Neither AICAR, A23187, nor A769662 had any effect on ADP:ATP ratios or basal or DNP-stimulated oxygen uptake in WT or RG cells, confirming that they do not inhibit mitochondrial metabolism. AICAR also failed to cause activation or phosphorylation of the R531G mutant, but this is consistent with previous studies showing that ZMP (the AMP mimetic formed by intracellular metabolism of AICAR) binds to the AMPK γ subunits in a similar manner to AMP (Day et al., 2007).

With the above exceptions (as well as 2-deoxyglucose, which inhibits glycolysis) we found that most compounds tested inhibit mitochondrial ATP production and thus activate AMPK indirectly by altering cellular AMP:ATP ratios. This included the classical mitochondrial inhibitors oligomycin and DNP; the guanidine derivatives metformin, phenformin, and galegine; the thiazolidinedione troglitazone; the barbiturate phenobarbital; and the phytochemicals resveratrol, quercetin, and berberine. These agents caused phosphorylation and activation of AMPK only in WT and not in RG cells, inhibited cellular oxygen uptake, and (apart from metformin) significantly increased ADP:ATP ratios. Except for phenobarbital and galegine, most of these agents have already been reported to inhibit the function of isolated mitochondria. Thus, metformin, phenformin, thiazolidinediones, and berberine inhibit respiratory chain complex I (El-Mir et al., 2000; Owen et al., 2000; Brunmair et al., 2004; Turner et al., 2008), while resveratrol and quercetin inhibit the ATP synthase (Zheng and Ramirez, 2000). After maximal inhibition of the respiratory chain, oxygen uptake should not be stimulated by the uncoupler DNP, and this was the case for metformin, phenformin, galegine, troglitazone, phenobarbital, and berberine. Conversely, the reduced oxygen uptake caused by drugs that inhibit the ATP synthase should be increased by DNP (which uncouples the respiratory chain from ATP synthesis), and this was observed with oligomycin and quercetin, and to a lesser extent with resveratrol.

Although quercetin has been found to inhibit the ATP synthase in isolated mitochondria (Zheng and Ramirez, 2000), our results suggest that it is not acting solely via this mechanism in intact cells. Quercetin produced a small but significant increase (1.6-fold) in ADP:ATP at 100 μM (Figure 5B) and a much larger increase at 300 μM (18-fold and 33-fold in WT and RG cells, data not shown). However, any decrease in oxygen uptake was undetectable at 100 μM and was quite modest (about 25%, Figure 6B) at 300 μM. These results suggest that at these concentrations quercetin may also be depleting ATP by another, unknown mechanism. Although the effect was much smaller than in WT cells, there was also a residual AMPK activation by quercetin in RG cells, indicating an additional AMP-independent effect.

Although metformin did not produce significant increases in ADP:ATP ratio, the fact that it caused phosphorylation and activation of AMPK in WT but not RG cells clearly shows that it was acting by increasing cellular AMP. We (Hawley et al., 2002) and others (Fryer et al., 2002) previously failed to detect a change in AMP:ATP ratio in cells treated with metformin. However, perfusion of intact rat heart with metformin was found to increase AMP in concert with AMPK activation (Zhang et al., 2007). In that study, AMP concentration was not measured in extracts but was calculated from the intracellular concentrations of phosphocreatine and ATP estimated by ^31^P-NMR, assuming that the creatine kinase and adenylate kinase reactions were at equilibrium. AMP is compartmentalized between mitochondria and the cytoplasm and may not be evenly distributed even within the cytoplasm (Dzeja and Terzic, 2003). The method used by Zhang et al. (2007) may estimate changes in the relevant pool of AMP more reliably than measurements in cell extracts. Our study demonstrates that a failure to detect a change in the levels of adenine nucleotides in a cell extract, whether in response to metformin (Fryer et al., 2002; Hawley et al., 2002) or resveratrol (Suchankova et al., 2009), does not provide conclusive evidence that the treatment is working via an AMP-independent mechanism.

Others have suggested more complex mechanisms by which metformin, resveratrol, and quercetin activate AMPK. It has been proposed that activation by metformin involves generation of reactive nitrogen species and a cascade involving c-Src and phosphatidylinositol 3-kinase (Zou et al., 2003), and/or by phosphorylation of LKB1 by protein kinase C-ζ, causing its translocation from the nucleus to the cytoplasm (Xie et al., 2008). It has also been proposed that resveratrol and quercetin activate the NAD-dependent histone deacetylase SIRT1, triggering deacetylation of LKB1 and phosphorylation of AMPK (Suchankova et al., 2009). However, many aspects of these hypotheses remain unconfirmed: even whether resveratrol activates SIRT1 directly (Howitz et al., 2003) has been challenged (Pacholec et al., 2010). It has recently been proposed that its effects on SIRT1 in vivo may be secondary to AMPK activation (Canto et al., 2009, 2010), and consistent with this, the metabolic effects of dietary resveratrol are lost in AMPK knockout mice (Um et al., 2010). Resveratrol inhibits the mitochondrial ATP synthase (Zheng and Ramirez, 2000) by binding to the γ subunit (Gledhill et al., 2007), and our results support the idea that it activates AMPK via this mechanism.

A surprising finding was that metformin caused a rather severe inhibition of the respiratory chain in HEK293 cells (Figure 6B), but caused only a modest activation of AMPK without significant changes in ADP:ATP. However, a key difference with the metformin experiments was the 16 hr incubation rather than the 1 hr used with all other treatments, a modification necessitated by the slow uptake of metformin into HEK293 cells (Shu et al., 2007). This prolonged incubation may give the cells more time to adapt to inhibition of oxidative phosphorylation, perhaps by upregulating glycolysis. The experiments in Figures 6 show that inhibition of the respiratory chain in H4IIE cells was almost maximal after 2 hr, as was AMPK activation. In these cells, AMPK activation by metformin was blocked by quinidine, a competitive inhibitor of OCT1. Interestingly, AMPK activation in response to phenformin or galegine was not blocked by quinidine, indicating that their cellular uptake is not dependent on the transporter. This is also consistent with our findings that these agents activated AMPK much more rapidly than metformin in HEK293 cells.

Overall, perhaps the most striking finding to emerge from our study was that many AMPK activators are inhibitors of mitochondrial function. Why should such a wide variety of xenobiotics inhibit mitochondria? One explanation is that the respiratory chain and ATP synthase are composed of several large, hydrophobic multiprotein complexes, potentially providing many binding sites where hydrophobic xenobiotics might inhibit their function. Another interesting possibility is that many of the AMPK activators are secondary plant metabolites and may have evolved as inhibitors of mitochondrial function to render the plants toxic to grazing animals or microbial pathogens. Interestingly, one of the common English names of *Galega officinalis* (from which galegine is derived) is goat's rue, signifying that the plant is poisonous to herbivores.

Our results also clarify the mechanisms of some other AMPK-activating treatments. First, hydrogen peroxide caused activation and phosphorylation of AMPK in WT but not in RG cells, increased the ADP:ATP ratio, and inhibited whole-cell oxygen uptake with no effect of subsequent DNP addition. Thus, although oxidative stress does activate AMPK (Choi et al., 2001; Hwang et al., 2005), the target for reactive oxygen species may not be AMPK itself but component(s) of the respiratory chain, leading to a secondary effect on AMPK via increases in AMP:ATP ratio. Second, our results are consistent with the idea that 2-deoxyglucose acts by inhibiting glycolysis, because it caused phosphorylation and activation of AMPK in WT but not RG cells and increased cellular ADP:ATP ratios but did not affect oxygen uptake. Third, osmotic stress using sorbitol appears to activate AMPK by multiple mechanisms. While it caused activation of AMPK in RG cells, this was significantly less than that observed in WT cells. It caused a significant increase in cellular ADP:ATP ratio and a decrease in basal oxygen uptake, but it also triggered intracellular Ca^2+^ release, and its effects were partially blocked by STO-609. Taken together, these results suggest that osmotic stress acts via two mechanisms, involving increases in both AMP and Ca^2+^.

An important subsidiary finding of our study was that, although the expression levels of the WT and R531G mutants of AMPK in the stably transfected cells were identical, the RG mutant was about twice as active when measured in the absence of AMP, associated with a 2-fold higher basal Thr-172 phosphorylation (Figure 1). While an increase in basal activity of the γ2 mutations has been previously proposed, this was either based on indirect assays after expression in yeast (Arad et al., 2002) or on kinase assays after transient transfection, which is complicated by variable expression levels (Burwinkel et al., 2005). In the stably transfected, isogenic cell lines used in this study, the size of the effect could be quantified in a more reliable manner. The RG mutant, despite its increased basal phosphorylation, was further activated by treatments that increased cytoplasmic Ca^2+^, but not by treatments that increased cellular AMP. We have shown previously that this mutation interferes with the binding to the γ2 subunit not only of the activating ligand, AMP, but also of the inhibitory ligand, ATP (Scott et al., 2004). This is consistent with structural studies of γ1 showing that the side chain of Arg-298 (equivalent to Arg-531 in γ2) is directly involved in binding of AMP and ATP to the exchangeable site formed by CBS repeats 3 and 4 (Xiao et al., 2007). Since AMP binding inhibits dephosphorylation of Thr-172, an interesting possibility is that ATP binding might enhance it. According to this model, the phosphorylation state of AMPK in unstressed WT cells is low because the majority of the complexes have ATP, rather than AMP, bound to the γ subunit, thus promoting dephosphorylation. However, due to reduced affinity for ATP, AMPK in unstressed RG cells might be partially nucleotide-free, causing enhanced net phosphorylation. Whatever the explanation, the RG mutation causes both loss of function (failure to be activated by AMP) and gain of function (increased basal activity). The gain-of-function effect explains not only why the genetic disorders in humans with R531G (or related mutations) are dominant but also why they are associated with increased glucose uptake and glycogen accumulation (Luptak et al., 2007).

A second subsidiary finding from our study was that, for most of the pharmacological agents tested, the increases in ADP:ATP ratio were larger in the WT than in the RG cells (Figure 5). One possible explanation is that the high basal activity of AMPK in the RG cells causes long-term adaptations that allow the cells to better withstand the metabolic stresses imposed, although we could not find any evidence that this involves increased mitochondrial biogenesis.

In summary, the use of WT and RG cells allows us to distinguish several different mechanisms by which AMPK can be activated, of which the first three cause indirect activation by increasing cellular AMP: (1) inhibition of the respiratory chain (biguanides, galegine, troglitazone, phenobarbital, berberine), (2) inhibition of the mitochondrial ATP synthase (oligomycin, resveratrol), (3) inhibition of glycolysis (2-deoxyglucose), (4) increasing cytoplasmic Ca^2+^ (A23187), (5) intracellular conversion into an AMP mimetic (AICAR), and (6) direct binding to AMPK at a site distinct from the AMP site (A769662). Some treatments, such as osmotic stress or quercetin, may work through more than one of these mechanisms.

## Experimental Procedures

### Materials and Proteins

STO-609 was from Tocris Bioscience, Bristol, UK. Phenformin, metformin, H_2_O_2_, sorbitol, DNP, A23187, phenobarbital, quercetin, resveratrol, berberine, and 2-deoxyglucose were from Sigma, Poole, UK. Oligomycin, troglitazone, and AICAR were from CN Biosciences, Nottingham, UK. Zeocin and hygromycin B were from Invitrogen, Renfrew, UK. Galegine was a generous gift from Brian Furman (Strathclyde Institute of Pharmacy and Biomedical Sciences University of Strathclyde, Glasgow, UK). A769662 was synthesized as described previously (Iyengar et al., 2005).

### Antibodies

The antibody against the phosphorylated form of Thr-172 on the AMPK α subunits was from New England Biolabs, Hitchin, UK. Antibodies against the *myc* and FLAG epitopes were from Sigma, Poole, UK. Antibodies against AMPK-γ1 were from Abcam, Cambridge Science Park, Cambridge, UK. Antibodies against AMPK-α1 and -α2 (Woods et al., 1996), AMPK β2 (Thornton et al., 1998), AMPK-γ2 (Cheung et al., 2000), and pACC (Hawley et al., 2003) were as described previously. Antibodies against AMPK-β1 were raised using the peptide KTPRRDSSGGT (residues 18–28 of rat sequence) with an N-terminal cysteine residue to allow coupling via the thiol to keyhole limpet haemocyanin. The immunization schedule and affinity purification of the antibody on a phosphopeptide column was as previously described (Sugden et al., 1999).

### Plasmids

Full-length human γ2 was amplified with primers designed to encode a 5′-BamHI site and a C-terminal FLAG tag followed by an XhoI site. The resulting PCR product was cloned into the pcDNA5/FRT plasmid (Invitrogen) to create pcDND5/FRT/γ2. Mutagenesis of the construct was performed using the QuikChange Site-Directed Mutagenesis system (Stratagene). The plasmid, POG44, expressing Flp recombinase was from Invitrogen.

### Generation of Stable Cell Lines

HEK293 cells containing a single FRT site (Invitrogen) were transfected with Lipofectamine 2000 (Invitrogen) using the plasmids POG44 and pcDND5/FRT/γ2 at a ratio of 9:1. Fresh medium was added to the cells 24 hr after transfection, and media containing 200 μg/ml hygromycin B was added 48 hr after transfection. The medium was replaced every 3 days until foci could be identified, and individual foci were then selected and expanded. The expression of FLAG-tagged γ2 was checked using immunofluorescence microscopy and Western blotting using anti-FLAG antibodies.

### Cell Culture

Flp-In HEK293 cells (Invitrogen) containing a single FRT site were cultured in Dulbecco's modified Eagles's medium (DMEM) containing 4.5 g/L glucose, 10% (v/v) fetal bovine serum (FBS), 100 IU/ml penicillin, 100 μg/ml streptomycin, and 100 μg/ml zeocin. After transfection, Flp-In HEK293 cells stably expressing either WT or R531G AMPK-γ2 were cultured as above except that zeocin was replaced by 200 μg/ml hygromycin B. After various treatments as indicated in the figure legends, cells were rinsed rapidly with ice-cold phosphate-buffered saline to remove the medium and then lysed in situ using 0.3 ml of ice-cold lysis buffer (50 mM Tris/HCl [pH 7.2], 1 mM EGTA, 1 mM EDTA, 50 mM NaF, 1 mM Na pyrophosphate, 1% [w/v] Triton X-100, 0.1 mM phenylmethane sulphonyl fluoride [PMSF], 1 mM DTT, 0.1 mM benzamidine, and 5 μg/ml soybean trypsin inhibitor). The lysates were centrifuged (4°C, 10 min, 21,000 × g) and the supernatants frozen for later analysis. H4IIE cells (provided by Dr. Calum Sutherland, University of Dundee) were grown as for HEK293 cells except that FBS was at 5% (v/v).

### Immunoprecipitate Kinase Assays of AMPK

Lysates containing stably expressed, recombinant FLAG-tagged γ2 (either WT or R531G) were immunoprecipitated from HEK293 cell lysates (90 μg protein) by incubation at 4°C for 2 hr on a roller mixer with 9 μl of EZview Red anti-FLAG M2 affinity gel (Sigma). After extensive washing, the immunoprecipitates were assayed for AMPK activity as described (Towler et al., 2008) using the *AMARA* peptide (Dale et al., 1995) as substrate, except for Figure 1C, where the *SAMS* peptide (Davies et al., 1989) was used.

### Estimation of Cellular ADP:ATP Ratio

Culture medium was quickly aspirated from cells grown on 6 cm culture dishes, and cells were washed with 1 ml of ice-cold PBS. After rapid aspiration of PBS, a minimal volume of ice-cold 5% perchloric acid was added and the samples vortexed to ensure complete lysis. After centrifugation (14,000 rpm, 4°C, 3 min) to remove acid-insoluble material, the supernatant was extracted with two washes of an equal volume of 1:1 tri-n-octylamine and 1,1,2-trichlorotrifluoroethane. The nucleotides remaining in the aqueous phase were then separated by capillary electrophoresis with on-column isotachophoretic concentration, using run buffers consisting of 50 mM Na phosphate and 50 mM NaCl (pH 5.2; leading buffer) and 100 mM MES/Tris (pH 5.2; tailing buffer). To each buffer was added 0.2% hydroxyethylcellulose to decrease electro-osmotic flow. Nucleotide peaks were detected by UV absorbance at 260 nM and integrated using System Gold software (Beckman). Nucleotide ratios were calculated from peak areas after correction for retention times. Identification of peaks as ATP, ADP, and AMP were confirmed by additional runs spiked with internal standards and analysis of absorbance spectra of individual peaks.

### Calcium Imaging

HEK293 cells were plated onto sterile 35 mm glass-bottomed tissue culture dishes (FluoroDish; World Precision Instruments, FL, USA) that were prepared by coating the glass base of each dish with 1 ml of 100 μg/ml poly-D-lysine hydrobromide (PDL) for 5 min. Cells were incubated for 30 min with 5 μM Fura-2-AM in PSS of the following composition (mM): 130 NaCl, 5.2 KCl, 1 MgCl_2_, 1.7 CaCl_2_, 10 glucose, and 10 HEPES (pH 7.4). After washing with Fura-2 free PSS for at least 30 min prior to experimentation, culture dishes were placed on a Leica DMIRBE inverted microscope. Cytoplasmic Ca^2+^ concentration was then reported by the Fura-2 fluorescence ratio (F340/F380 excitation; emission 510 nm) as previously described (Calcraft et al., 2009). Emitted fluorescence was recorded at 22°C with a sampling frequency of 0.1 Hz, using a Hamamatsu 4880 CCD camera via a Zeiss Fluar 40 × , 1.3 n.a. oil immersion lens, and Leica DMIRBE microscope. Background subtraction was performed online. Analysis was via Openlab imaging software (Perkin-Elmer, UK).

### Other Analytical Procedures

SDS/PAGE was performed using precast Bis-Tris 4%–12% gradient polyacrylamide gels in the MOPS buffer system (Invitrogen). Proteins were transferred to nitrocellulose membranes (Bio-Rad) using the Xcell II Blot Module (Invitrogen). Membranes were blocked for 1 hr in Odyssey blocking buffer (Li-Cor biosciences, Cambridge, UK). The membranes were probed with appropriate antibody (0.1–1 μg/ml) Odyssey blocking buffer. Detection was performed using secondary antibody (1 μg/ml) coupled to IR 680 or IR 800 dye and the membranes scanned using the Li-Cor Odyssey IR imager. Protein concentrations were determined by Coomassie blue binding (Bradford, 1976) with BSA as standard.

### Measurement of Whole-Cell Oxygen Uptake

Oxygen uptake was measured in 24 well plates using the Seahorse XF24 Extracellular Flux Analyzer. Cells were incubated at a density of about 10^6^ cells per well in unbuffered medium (pH 7.4): 8.3 g/L DMEM base, 200 mM GlutaMax-1, 100 mM Na pyruvate, 25 mM glucose, 32 mM NaCl, and 40 μM Phenol Red. Oxygen consumption rates were continuously measured as described previously (Wu et al., 2007).

### Statistical Analysis

Statistical analyses, as detailed in the figure legends, were performed using GraphPad Prism 5 for Mac OSX.

## Supplemental Information

Document S1. Supplemental Results and One Figure
