# Supplementary material for: Use of Cells Expressing γ Subunit Variants to Identify Diverse Mechanisms of AMPK Activation
Source: Cell Metab. 2010 Jun 9;11(6):554–65. doi: 10.1016/j.cmet.2010.04.001 (PMC2935965; doi:10.1016/j.cmet.2010.04.001)
Supplement: Document S1. Supplemental Results and One Figure [file mmc1.pdf]

Cell Metabolism, Volume *11*

Supplemental Information

Use of Cells Expressing γ Subunit Variants to Identify Diverse Mechanisms of AMPK Activation

Simon A. Hawley, Fiona A. Ross, Cyrille Chevtzoff, Kevin A. Green, Ashleigh Evans, Sarah Fogarty, Mhairi C. Towler, Laura J. Brown, Oluseye A. Ogunbayo, A. Mark Evans, and D. Grahame Hardie

Supplemental Results

Measurements of oxygen uptake in RG cells, obtained under identical conditions to those in WT cells Fig. 6A and 6B, are displayed in Figure S1.

Hawley et al Figure S1

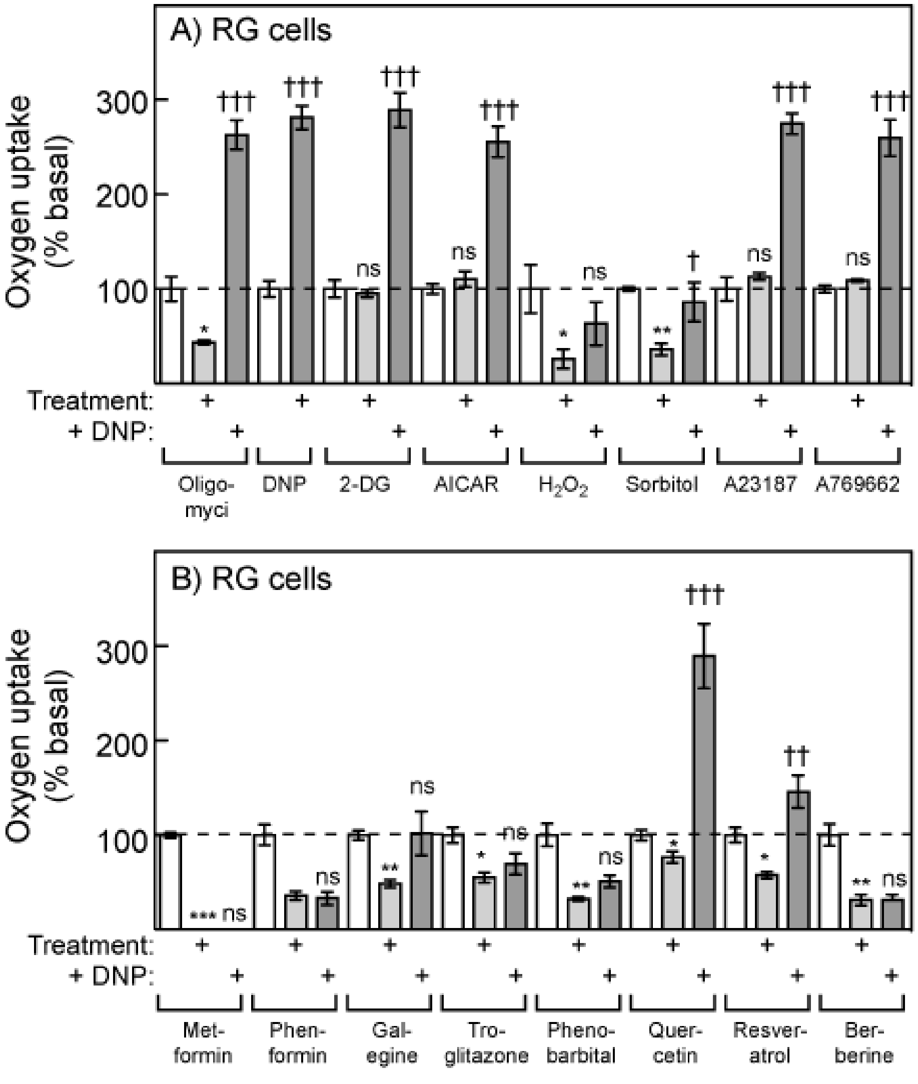

Figure S1A/B: Effect of various agents on oxygen uptake in RG cells. Experiments were exactly as for as for Fig. 6A-B except that RG cells replaced WT cells.
